# Supralinear scaling behavior of ionic transport in membrane nanochannels regulated by outer-surface charges†

**DOI:** 10.1039/d4na00540f

**Published:** 2024-10-14

**Authors:** Laidy M. Alvero-González, Marcel Aguilella-Arzo, D. Aurora Perini, Lucie A. Bergdoll, María Queralt-Martín, Antonio Alcaraz

**Affiliations:** a Laboratory of Molecular Biophysics, Department of Physics, University Jaume I 12071 Castellón Spain mqueralt@uji.es alcaraza@uji.es; b Instituto de Ciencia Molecular, Universidad de Valencia Catedrático José Beltrán-2 46980 Paterna Spain; c Laboratoire d'Ingénierie des Systèmes Macromoléculaires, CNRS – Aix Marseille Université 31 Chemin Joseph Aiguier Marseille France

## Abstract

The peculiarity of ion transport at the nanoscale is revealed through electrophysiological studies of two biological ion channels: the cation-selective bacterial porin-OmpF and the mitochondrial voltage-dependent anion channel (VDAC). We provide evidence of an unprecedented scaling behavior in the power-law relationship between conductivity and concentration *G* ∼ *c*^*α*^ with *α* > 1 when functional groups attached to the pore inner wall have opposite charges to those located in the nanochannel's outer surface. Indeed, we find *α* ∼ 1.4 both for OmpF in positively charged membranes and for VDAC in negatively charged ones. The experiments are analyzed using different levels of theoretical models, starting with an equivalent circuit where total electrical current is described as the sum of ionic currents. Subsequently, we show that electrical circuits incorporating simplifying assumptions such as local electroneutrality and Donnan equilibrium consistently account for the measured *G*–*c* relationships yielding extremely similar results to the numerical results of structure-based Poisson–Nernst–Planck equations computed without these assumptions. We demonstrate that unexpected scaling exponents do not correspond to deviations from these classical equilibrium/electroneutrality assumptions, but rather to the structural features of the pore that are not included in oversimplified models in terms of shape and/or charge distribution. In contrast to the predictions of widely accepted models, we demonstrate both experimentally and theoretically that the conductance of ion-selective nanochannels can be drastically reduced in dilute solutions through a mechanism in which membrane charges and pore charges do not compensate for each other but act as interacting sites of opposite charge. Our insights into the critical role of external surface charges aim to open new conceptual avenues for developing nanofluidic devices with enhanced capabilities for energy conversion and sensing properties.

## Abstract 2

Supralinear scaling is found when functional groups attached to the pore inner wall have opposite charges to those located in the nanochannel's outer surface.

## Graphical abstract

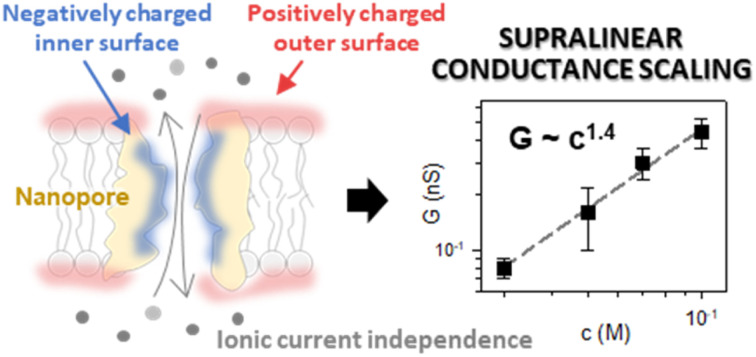

## 1. Introduction

Ion transport through membrane channels has been the subject of intensive research in fields such as physical chemistry, electrochemistry, soft matter, nanomaterials, and biophysics, offering a variety of perspectives.^1–8^ While a general body of knowledge can support discoveries in all these interrelated fields by invoking basic concepts like Debye screening, Poisson–Boltzmann electrostatics or Donnan equilibrium,^2,9^ unexpected behaviors are also detected when pore dimensions approach molecular sizes. Highly confined nanometric channels (ranging from single-digit nanopores to angstrom-sized pores) exhibit surprising phenomena such as electroneutrality breakdown,^10^ anomalous dielectric behaviors,^11^ interfacial effects^12,13^ or overlapping electric double layers.^14^ Understanding which nanoscale properties cause the deviations from paradigms that work well at the microscale is essential for designing atomic-sized fluidic devices required for new sensing technologies, ionic circuits, iontronic components, radioactive-ion sieving, photonics, energy conversion, chemical separation and desalination, among others.^3,15–19^ This knowledge is also critical for understanding how biological ion channels function and predict their properties under various physiological conditions that are not always accessible for experimental validation.^20^

Among the transport properties that could potentially be investigated, the so-called concentration scaling behavior, *i.e.*, the power-law relationship between channel conductance and electrolyte concentration *G* ∼ *c*^*α*^, has attracted particular attention because it allows a rapid rationalization of the observed experimental trends.^13,21–24^ So far, predicted and experimentally reported values for *α* are constricted to 0 ≤ *α* ≤ 1. Although *α* is a phenomenological exponent, its physical origin is the subject of intensive investigation because different factors influence its value, including ion accumulation due to charged surfaces or interfacial effects, among others.^7,9,12,13,21,23,25–29^

Although at this moment there is consensus that exponents 0 ≤ *α* ≤ 1 arise from a balance between surface, bulk and interfacial effects, there is no general agreement on how to assess the importance of each one of these elements.^4,12,13,21,22,30–33^ Also, there is intense debate on which particular theoretical and/or computational approach is more appropriate to explain the experimental results, ranging from simple equivalent circuits to continuum approaches.^9,12,34^ In the latter, there are different alternatives that include charge regulation,^25,35^ the combination of pore conduction and interfacial effects governed by Donnan equilibrium^12,25^ or by electroneutrality breakdown inducing a leakage of surface potential into the solution.^10,21^ Alternatively, structure-based Poisson–Nernst–Planck calculations performed without electroneutrality requirements or Donnan equilibrium^13^ and Molecular Dynamics (MD) simulations restricted to physiological and concentrated solutions^36^ are available.

Within this context, here we first report experimental *G vs. c*^*α*^ curves with *α* > 1 in different systems and under variable conditions, providing robust evidence of unprecedented supralinear scaling. We attain *α* > 1 by using biological nanochannels embedded in lipid membranes, where we can separately modify lipid and channel charges. By experimentally tuning system conditions, we determine that the supralinear scaling arises from non-neutralizing opposite constellations of charges at the channel entrances and pore interior. Next, we use the experimental results to test available theoretical and computational models and discuss the validity of the assumptions on which they are based. This includes equivalent circuit models and various iterations of structure-based 3D continuum computations. By comparing model predictions and experiments, we demonstrate that the simplest possible model – an equivalent circuit with resistors – is adequate to describe conductance scaling if it includes the assumption of ionic current independence. Yet, more advanced structure-based calculations including continuum computations are used to confront the experimental data and test previously questioned assumptions such as electroneutrality or equilibrium.^9,10,21^ We demonstrate, on the one hand, that intermediate scaling exponents 0 ≤ *α* ≤ 1 and/or 1 ≤ *α* ≤ 2 do not correspond to deviations from classical equilibrium/electroneutrality assumptions, but rather to the structural features of the pore that are not included in oversimplified models, such as non-cylindrical shape, inhomogeneous charge distribution or entrance effects. On the other hand, we support the hypothesis that supralinear scaling arises from the existence of non-neutralizing opposite charges in the system.

Overall, the present work advances our understanding of nanoscale ion transport by, first, providing a set of measurements with groundbreaking *G*–*c* relationships that are incompatible with current theoretical models, and second, presenting a new framework that successfully rationalizes the interplay between inner- and outer-surface charges in membrane nanochannels while preserving the traditional interpretation of previous results. Our findings are especially significant for the permeability of biomembranes, traditionally understood mainly in terms of cellular expression of protein channels tightly gated by specific bioelectrochemical stimuli such as voltage or ligands.^37^ We hypothesize that the conductance of open channels may be significantly reduced by subtle electrochemical mechanisms based on interaction between matching clusters of opposite charge,^38^ namely lipid charges and proper channel charges. Our results also suggest that biological channels reconstituted in tailor-made membranes could be useful for nanofluidic device development based on the independent functionalization of inner and outer channel surfaces,^39,40^ with promising impacts for energy conversion and enhanced biosensing properties.^41^

## 2. Materials and methods

### 2.1. Protein production and purification

Wild-type OmpF was a kind gift of Dr S. M. Bezrukov (NIH, Bethesda, MD, USA) and the OmpF mutant with residues D113 and E117 replaced with arginine (OmpF RR) was a kind gift of Dr H. Miedema (Wetsus, The Netherlands).^42,43^

Recombinant mouse VDAC1 was produced and purified as described previously.^44^ In brief, the mouse VDAC1 gene bearing a 6-his tag in the N-terminus inserted into the pQE9 vector was transformed into M15 *E. coli* cells for protein expression. Cells were grown at 37 °C in the LB medium to *A*_600_ = 0.8 and induced with 0.4 mM IPTG for 4 h. Cells were harvested and resuspended in 50 mM Tris–HCl (pH 8.0), 2 mM EDTA, 20% sucrose, 0.6% Triton X-100, and 0.1 mg mL^−1^ lysozyme. The resuspended pellet was sonicated and centrifuged (12 000×*g*, 15 min) to obtain inclusion bodies. The inclusion body pellet was washed with wash buffer [20 mM Tris–HCl (pH 8.0), 300 mM NaCl, and 2 mM CaCl_2_], repelleted and solubilized in equilibration buffer [20 mM Tris–HCl (pH 8.0), 300 mM NaCl, and 8 M urea]. VDAC1 was purified from solubilized inclusion bodies using a Ni-NTA metal affinity column, equilibrated in equilibration buffer, washed with equilibration buffer containing 30 mM imidazole and eluted with equilibration buffer containing 150 mM imidazole. The pure protein was transferred to a 7 kDa dialysis bag and refolded by dialysis in two steps: (i) 20 mM Tris–HCl (pH 8.0), 300 mM NaCl, 1 mM DTT, and 4 M urea; 1% LDAO was added to the dialysis bag and transferred to dialysis buffer; (ii) 20 mM Tris–HCl (pH 8.0), 300 mM NaCl, and 1 mM DTT. Refolded protein was ultracentrifuged (355 000×*g*, 30 min) to remove aggregated protein and concentrated by using an Amicon Ultra-50 (Millipore). The refolded protein sample was applied to a Superdex 200 column and eluted with SEC buffer [20 mM Tris–HCl (pH 8.0), 150 mM NaCl, 1 mM DTT, and 0.1% LDAO] to obtain a homogeneous protein population. The refolded peak was stored at −80 °C after the addition of 20% glycerol.

### 2.2. Membrane formation, channel reconstitution and electrical measurements

The OmpF and VDAC channel measurements were carried out by reconstituting a single protein into a planar lipid membrane using the modified Montal–Mueller technique.^45,46^ Membranes were prepared from neutral diphytanyl phosphatidylcholine (PC), negatively charged diphytanyl phosphatidylserine (for OmpF) or dioleoyl phosphatidylserine (for VDAC) (PS) or positively charged dioleoyl trimethylammonium propane (TAP) either pure (for OmpF) or mixed with PC at a ratio of 2 : 1 (w/w) (for VDAC). All lipids were purchased from Avanti Polar Lipids. Aliquots of 10–20 μL of 5 mg mL^−1^ of lipids were added on top of each salt solution in two 1.8 mL compartments (so-called *cis* and *trans*) of a Teflon chamber. The two compartments were separated by a 15 μm-thick Teflon film with a 70–100 μm diameter orifice where the membrane is formed. The orifice was pre-treated with a 3% solution of hexadecane in pentane. After pentane evaporation, the level of solutions in each compartment was raised above the hole so a planar bilayer could form by apposition of the two monolayers. The chambers were filled with a solution of 0.03–1.5 M KCl buffered with 5 mM HEPES at pH 7.4 for VDAC essays and 0.003–1.5 M KCl buffered with 5 mM HEPES at pH 6 for OmpF measurements. Membrane formation was tracked by applying a triangular voltage wave and visualizing the output current using an oscilloscope. Because a sealed membrane acts as a capacitor, a zero-averaged output current with a perfect square shape manifests the formation of a stable non-leaking membrane. From the pic-to-pic current amplitude, the membrane capacitance can be inferred. After membrane formation, voltages of different amplitudes from ±10 to ±200 mV were applied to verify that the membrane was not leaking. Only membranes displaying zero current under these voltage pulses were used for subsequent channel insertion. At low salt concentrations, stable membranes required the addition of more lipid (up to 25–30 μL per side) compared to the experiments at high salt concentrations, regardless of the lipid used to form the membrane.

VDAC channel insertion was achieved by adding 0.5 μL of VDAC1 diluted at 0.003 mg mL^−1^ in buffer containing 10 mM Tris, 50 mM KCl, 1 mM EDTA, 15% (v/v) DMSO, and 2.5% (v/v) Triton X-100, pH 7.0, into the *cis* compartment and OmpF channel reconstitution was accomplished by adding 0.1 μL of OmpF protein at 1 ng mL^−1^ in 1 M KCl and 1% (v/v) Octyl POE (Alexis, Switzerland) to the *cis* side of the chamber. Protein insertion was detected by an increase in the measured current under an applied voltage of 10 mV (VDAC) or 100 mV (OmpF), a value below the typical threshold for the closure of these channels,^47,48^ so the current measured corresponds to the open state. The presence of a single protein channel in the membrane was validated in two ways: first, by measuring the open channel conductance *G* (*G* = *I*/*V*) and comparing it with the reported/expected value for the measured conditions. Second, by applying a high voltage (typically 50–60 mV for VDAC and 150–200 mV for OmpF) to observe channel closure. When there is a single channel, a closure event manifests as a single step-wise current reduction of ∼50% of open-channel current in VDAC^48^ and in three step-wise current drops of ∼33% of open channel current each in OmpF reflecting the trimeric character of this channel.^47^ To ensure reproducibility, the experiments were repeated a minimum of 3 times.

The membrane potential was applied using Ag/AgCl electrodes in 2 M KCl/1.5% agarose bridges assembled within standard 200 μl pipette tips. Potential is defined as positive when it is greater on the side of protein addition (*cis* side). Current recordings were performed using an Axopatch 200B amplifier (Axon Instruments, Inc.) in voltage-clamp mode. Data from the amplifier were filtered using an integrated low pass 8-pole Bessel filter at 10 kHz, digitized with a Digidata 1440A (Molecular Devices, Sunnyvale, CA) at a sampling frequency of 50 kHz and analyzed using pClamp 10.7 software (Molecular Devices, Sunnyvale, CA). The chamber and the head stage were isolated from external noise sources with a double metal screen (Amuneal Manufacturing Corp., Philadelphia, PA). The described set-up can measure currents on the order of picoamperes or above with a time resolution below one millisecond.^49,50^

### 2.3. Theoretical calculations: PNP-3D equations

The so-called Poisson–Nernst–Planck (PNP) equations are mean-field phenomenological equations that describe ion transport through ionic channels.^51^ The Poisson equation relates the position-dependent electric charge density to the electrostatic potential of the system, while the non-equilibrium ionic fluxes are calculated using the Nernst–Planck equations.^52–54^ The channel fixed charge is obtained from the atomic charges of the protein using the charmm36 force field parameters in the neutral pH configuration of our study, using the three-dimensional structure of the OmpF and VDAC channels (Protein Data Bank code: 2OMF for OmpF and 3EMN for VDAC). The numerical solution of the system equations has been obtained using FiPy in Python,^55^ a solver of partial differential equations as described in detail elsewhere.^53^ The excluded solvent region was defined by incorporating the CHARMM radius for each protein atom, with an added solvent radius of 1.2 Å. This approach ensures the exclusion of both protein atoms and their immediate solvation shell from the solvent region. Van der Waals radii was used to to distinguish between regions of different permittivities, adopting only two dielectric values in our model: one for the solvent (*ε*_W_ = 78) and one for the protein/membrane region (*ε*_P_ = 2). For membrane lipids, a low dielectric region was modeled around the protein that is inaccessible to both solvent and ions. In the case of charged lipids, two constant charge regions of 5 Å depth were added near the solvent interface to simulate the charged lipid headgroups. Each region corresponded to a surface charge density of approximately 0.36 C m^−2^ (this amounts approximately to an elementary charge per each ∼44.2 Å^2^). Although the depth of charge distribution had minimal effect on our results, selecting a depth that is too small could lead to numerical instabilities. Thus, the current algorithm includes neither ion sizes nor charge regulations, as doing so would significantly increase computational effort. Boundary conditions were set as zero flux (Neumann) on the sides of the simulation box (the sides in contact with the membrane/protein system), and Dirichlet boundary conditions were used to specify potentials and concentrations at the top and bottom of the box (sides away from the protein/membrane system). These boundary conditions ensure a controlled electrostatic environment and species distribution and reproduce the experimental setup with good accuracy. The use of low concentrations in our computations required a box of sufficient length to ensure the relaxation of both concentrations and electrostatic potential as we move away from the membrane/protein system. The resulting system box had dimensions of approximately 585 × 585 × 565 Å for OmpF and 350 × 350 × 345 Å in the case of VDAC. Further details on the discretization methods and other parameters can be found elsewhere.^13,53,54^

## 3. Results and discussion

### 3.1. Experimental scaling of ion channel conductance

**Fig. 1:**
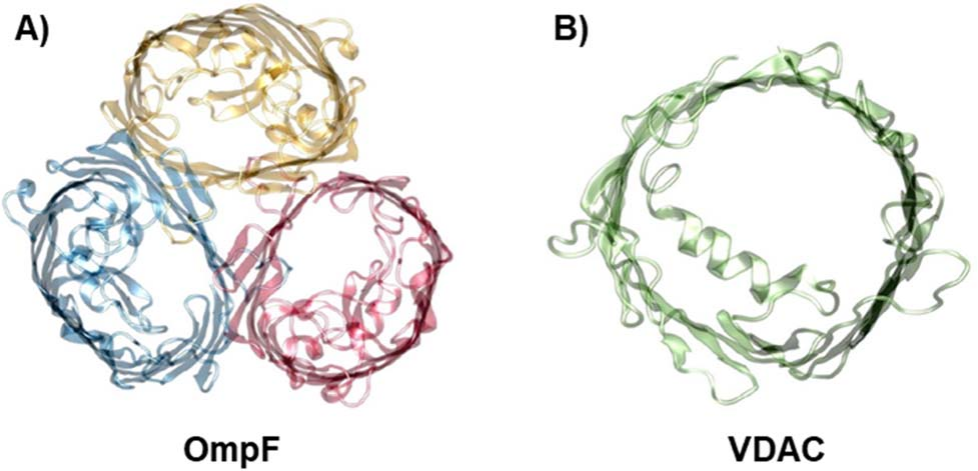
The biological nanopores OmpF and VDAC. Front view of the bacterial porin OmpF from *E. coli* (PDB code 2OMF) (A) and the mitochondrial VDAC (PDB code 3EMN) (B).

To investigate the influence of membrane composition on the channel conductance, we performed voltage-clamp electrophysiology in planar membranes in which the bacterial porin OmpF from *E. coli* (Fig. 1A)^47,56^ or the mitochondrial Voltage Dependent Anion Channel (VDAC) (Fig. 1B)^57^ was reconstituted. While OmpF is a cation selective channel, the VDAC is an anion selective pore, meaning that they have opposite net charges. Both channels are quite conductive, mildly selective and wide enough to allow the multi-ionic transport of hydrated ions, water and small solutes.^58^ To tune lipid membrane charge, we built phospholipid planar bilayers containing either neutral lipids (PC), negatively charged (PS) or positively charged ones (TAP).

**Fig. 2:**
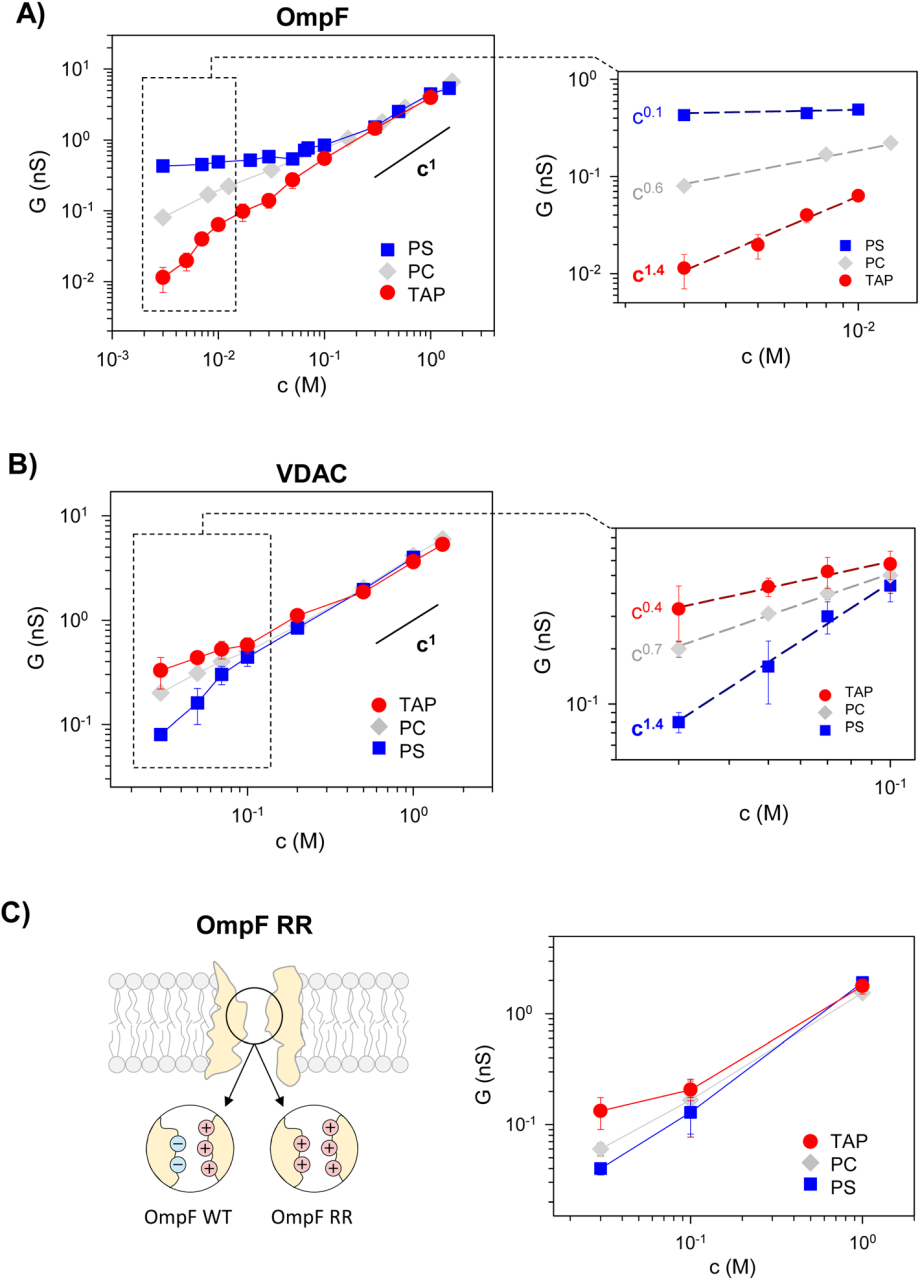
Membrane lipid charge modulates OmpF and VDAC conductance inducing supralinear scaling. Conductance *vs.* concentration curves of OmpF WT (A), VDAC (B), and the OmpF RR mutant (C) inserted in negatively (PS), neutral (PC) or positively (TAP) charged lipid membranes. The right panels in (A) and (B) show the scaling exponents at the low-concentration limit, attaining supralinear values for OmpF in TAP and VDAC in PS. Note that the data points used for the fittings range almost one order of magnitude in concentration.

Fig. 2 (left panels) displays conductance measurements in a wide range of KCl concentrations for OmpF (A) and VDAC (B) inserted in the differently charged lipid membranes, as labeled. In the high concentration regime (*c* > 100 mM) of both channels, *G* is independent of the lipid charge with scaling behavior interpreted as bulk-like behavior (*G* ∼ *c*^1^).^12,23,28^ Our results here agree with those found either in the high concentration limit of charged nanopores^23^ or in the whole curve of uncharged ones.^21^

In dilute solutions, the situation is different: lipid charge determines the overall conductance with a variety of scaling behaviors. Fig. 2A and B (right panels) show a zoomed-in view of the low concentration limit for a better observation of the scaling exponents. Since both channels are considerably ion-selective in dilute solutions regardless of membrane charge,^48,56,59,60^ a key role of pore charges in these conditions could be expected.^7,9,13,23^ However, the small scaling exponents characteristic of surface-governed processes^7,9,23,26,27^ are only found here for certain membrane compositions, namely *α* ∼ 0.1 for OmpF in PS and *α* ∼ 0.4 for VDAC in TAP. Higher scaling exponents that appear here in neutral membranes (*α* ∼ 0.6 and 0.7 in OmpF and VDAC, respectively) have been linked in previous studies to the contribution of access resistance and interfacial effects.^12,29,61^

Amazingly, both OmpF and VDAC show scaling exponents *α* > 1 (1.4 in both cases), which, to our knowledge, have never been reported experimentally or theoretically (so far, the reported values were 0 ≤ *α* ≤ 1). Here it is important to recall that OmpF is a trimer and VDAC is a monomer. Although the experimental evidence collected to date indicates that the three monomers of OmpF are structurally and functionally independent regarding channel conductance,^47,62,63^ one could speculate that the supralinear scaling found in OmpF is related to the loss of independence between monomers giving rise to a complex interplay not described before. However, this is not the case because experiments with VDAC, which is a monomeric channel, at the single channel level show also supralinear scaling.

Of note, values of *α* > 1 are found when the ion intrinsically preferred by the channel and the lipid membrane charge have the same sign (VDAC selective to anions/negative PS and OmpF selective to cations/positive TAP). To clarify this possibility, we extended the experiments to a mutant of the OmpF porin where two acidic residues (D113 and E117) in the central constriction have been replaced with arginines (OmpF RR, Fig. 2C). This mutant is significantly less conductive than the WT OmpF and, most important for the present study, selective to anions,^43,64^ meaning that the effective charge of the OmpF WT channel is reversed in OmpF RR. Fig. 2C (right panel) displays the conductance scaling of OmpF RR in neutral (PC), negatively charged (PS) or positively charged (TAP) membranes, showing that the *G*–*c* relationships in the different membrane compositions follow the same trends as the anion selective channel VDAC (Fig. 2B) and not those of the cation selective OmpF WT (Fig. 2A). Unfortunately, OmpF RR is too poorly conductive to obtain information about the actual scaling exponents in dilute solutions. However, it is clear from these results that the less conductive conformation occurs when the membrane charge and channel effective charge are opposite (negatively charged membrane and positively charged (anion selective) channel for VDAC and OmpF RR, and positively charged membrane and negatively charged (cation selective) pore for OmpF WT).

Therefore, we can conclude that supralinear scaling of conductance occurs in wide biological ion channels known as general diffusion porins (due to their lack of substrate-specificity^58,65–67^) only when the membrane charge and the channel effective charge have opposite signs. The possibility that this behavior could be a general feature of all nanometer-sized pores is still uncertain and probably would require a more extensive range of experimental conditions and possibly diverse types of channels and membranes. In fact, supralinear scaling has not been reported to date in synthetic nanopores, probably because the whole substrate typically has the same functionalization. Only recently, independent functionalization of inner and outer-surface charges has been explored.^39^

To further analyze the requirement of opposite charges and understand the implications of our experimental findings, we next compare the reported experimental data with different levels of theoretical interpretations.

### 3.2. Equivalent circuits to model ion permeation through nanometer sized pores

**Fig. 3:**
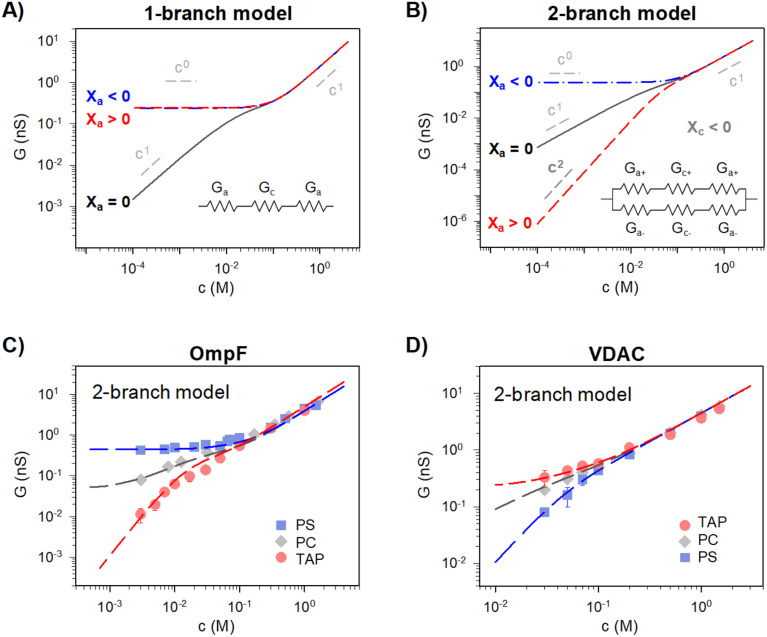
A 2-branch equivalent circuit model predicts supralinear scaling and fits the experimental data, while the 1-branch model does not. (A and B) Conductance scaling predictions of the 1-branch (A) and 2-branch (B) equivalent circuit models. The 1-branch model predicts a scaling relationship of *G* ∼ *c*^*α*^ with *α* = 0 for charged membranes regardless of their polarity. However, the 2-branch model returns different scaling behaviors for different membrane polarities, with *α* = 0 when channel and membrane charges have the same polarity (*X*_c_ < 0 and *X*_a_ < 0) and *α* = 2 when they are opposite (*X*_c_ < 0 and *X*_a_ > 0). Insets show the schematic representation of each circuit. The parameters used are radius *r* ∼0.5 nm and length, *L* ∼4 nm (aspect ratio *r*/*L* ∼ =0.125) and fixed charge density *X*_c_ ∼ 250 mM (values representative of a large variety of mildly cation-selective channels^56,68,69^) in KCl solutions (*D*_+_ = 1.95 × 10^−9^ m^2^ s^−1^ and *D*_−_ = 2.03 × 10^−9^ m^2^ s^−1^). When *X*_a_ ≠ 0 the value used was ±100 mM, representing characteristic values for membrane charges (see ref. 33 for a detailed explanation of how membrane charges are incorporated into continuum models). Also important, pore dimensions are wide enough to ensure that the application of mean field theories is meaningful.^13,52,58,70^ (C and D) Comparison of experimental conductance *vs.* concentration curves (solid points) and the 2-branch model (dashed lines) for OmpF (C) and VDAC (C) inserted in the differently charged lipid membranes, as indicated in the legend.

Traditionally, ion transport through nanometer sized pores has been described using a 1-branch equivalent circuit composed of three resistors in series^9,12,13,21^ as shown in the inset of Fig. 3A. The access conductance *G*_a_ (or its reciprocal, access resistance) in the channel mouth is connected in series with the channel proper conductance *G*_c_ and with the other *G*_a_ in the opposite channel mouth.

Then, the equivalent conductance *G* of the circuit in Fig. 3A is:1
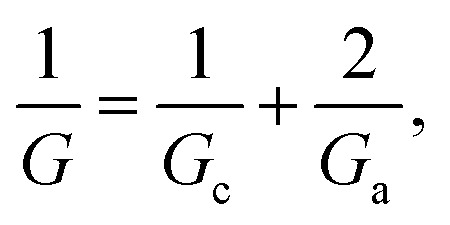
where *G*_c_ is the channel proper conductance and *G*_a_ corresponds to the access conductance. The 1-branch equivalent circuit is a simplification obtained considering that individual ionic contributions can be added in each of the regions. For *G*_c_, the simplest approximation is to consider that the channel is a solution-filled cylinder of radius *r* and length *L*:2
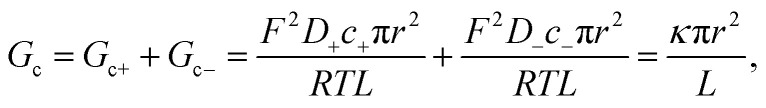
where constants *F*, *R*, and *T* have their usual meaning,^5^*D*_+_ and *D*_−_ represent the diffusion coefficients of cations and anions, respectively, *c*_+_ and *c*_−_ are the concentrations of cations and anions, respectively, and *κ* the conductivity of the full electrolyte defined as:3
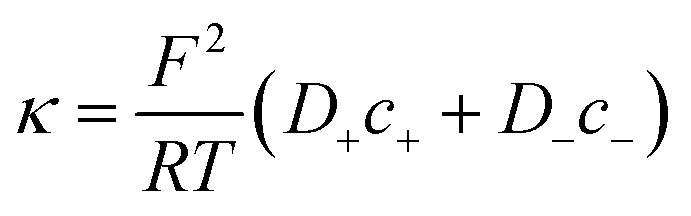


To calculate the contribution from “access conductance” *G*_a_, we use Hall's classical expression:^4,71^4
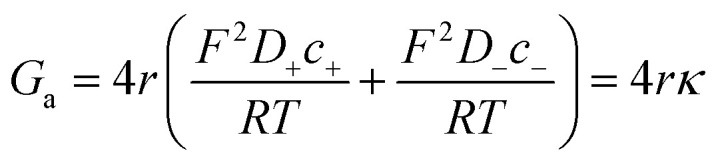


Eqn (2) and (4) correspond to a neutral pore embedded on a neutral membrane, so that no concentration gradients are expected to appear in the system (ionic concentrations inside the pore are identical to bulk ones) and ions are transported exclusively by electrical migration. However, charge effects have been shown to be crucial for both *G*_c_ and *G*_a_. On the one hand, a variety of experiments show a plateau in the *G versus c* curve at low ionic strength,^23,26^ not anticipated by eqn (1)–(4). Following the analogy with glass capillaries in microfluidics^72,73^ and the classical description of transport in ion-exchange membranes,^6,74^ it was suggested that *G*_c_ is actually given by the addition of two conducting regions: the bulk phase described by eqn (2) and a surface-governed-region where there is an excess of mobile counterion concentration arising from the charges located in the pore surface.^7,9,75^ This latter contribution can be included in eqn (2) using the Donnan formalism to calculate ionic concentrations inside the charged channel,^12,21,25,76^ so that *G*_c_ becomes:5
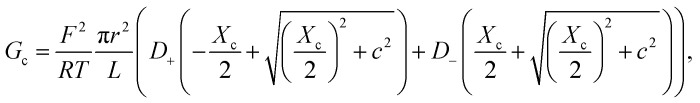
where *X*_c_ is the concentration of channel fixed charges.^5^ Note that *X*_c_ represents an effective one-dimensional average of the actual three-dimensional charge distribution inside the pore.^56,68^ Such idealization uses a “sponge model” that depicts the ion-exchange membranes as a heterogeneous system consisting of the inert matrix and the pore liquid.^4–6^

On the other hand, charges located near the pore entrance also determine access conductance *G*_a_ as reported in the OmpF bacterial channel^12^ and other ion channels like alamethicin, gramicidin A or the pores generated by the SARS-CoV-E protein.^13^ To account for these findings, Hall's original equation can be modified by considering again the Donnan formalism^76^ to explain how the local electrical conductivity is changed near a charged membrane (here represented by an effective fixed charge concentration *X*_a_), so that *G*_a_ turns into:^12,13^6
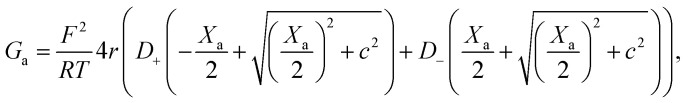


Interestingly, the effects of the charges located in the channel mouth (due to lipids or the pore itself) have also been described using the surface potential of Gouy–Chapman theory.^22,32^ Although both formalisms include different concepts and simplifying assumptions, Donnan (used in transport processes in membranes^76^) and Gouy–Chapman (common in colloid science^77^) lead to equivalent results when compared to the exact solution of the Poisson–Boltzmann equation.^78,79^ Note that the introduction of charge effects in eqn (5) and (6) implicitly assumes that ionic concentrations inside the membrane/channel system are different from bulk ones, turning the circuit of Fig. 3A into just an electrical analogy for an actual electrodiffusional transport governed by the gradient of electrochemical potential.

Eqn (1)–(6) (or slight modifications of them) have been quite successful in accounting for experiments carried out in synthetic^9^ and biological nanopores.^13^Fig. 3A shows typical *G* – *c* curves generated by the 1-branch model for a cation selective channel (*X*_c_ < 0) using different values of the membrane fixed charge *X*_a_ (see the caption of Fig. 3 for details about the representative values for the parameters). Irrespective of the sign of *X*_a_, the model predicts conductance saturation (*α* ∼ 0) in the low concentration limit linked to the accumulation of ions induced by membrane charges.^7,9,23,26,27^ In any other situation, a linear scaling (*α* ∼ 1) is predicted resembling bulk-like conductivity behavior^28^ (Fig. 3A): neutral systems,^21^ charged ones with dominating interfacial effects (access resistance)^12,29^ or in the high concentration limit of any system.^23^ In this latter case, charge effects are negligible and *G* ∼ *c*^1^ because the limit *c* ≫ *X*_c_ in eqn (5) and (6) leads to eqn (2) and (4), respectively.

However, these equations cannot describe the experimental data reported here (Fig. 2), where different scaling is observed in dilute solutions for different polarities of a charged membrane. In contrast, eqn (1)–(6) yield the same result (*G* ∼ *c*^0^) for any charged membrane, regardless of their sign (Fig. 3A for *X*_a_ > 0 and *X*_a_ < 0). Within the 1-branch model, both positive and negative membranes are equally effective in increasing the local conductivity at the channel mouths so that access effects appearing in the neutral membrane do not become dominant.

In view of the disagreement between the 1-branch model and the experiments reported in Fig. 2, it seems necessary to reexamine some fundamental assumptions made when building the model. A thought-provoking situation of the equivalent circuit depicted in Fig. 3A could be the case of an ideally selective pore flanked by non-selective access regions like a neutral membrane: coions and counterions contribute equally to the total current in the non-selective access region but their contributions become dramatically different within the channel limits. Within this view, individual ionic currents of a fully dissociated salt like KCl are neither continuous nor independent (even in extremely dilute solutions) given that they must combine precisely to give a position-independent overall current.

In contrast to the model depicted in Fig. 3A, many models for ion transport in membrane systems assume that cations and anions move independently when an electric potential is applied to the system, so that each ionic current is continuous and hence the total current is also continuous. For instance, in the most common form of Nernst–Planck formalism the flux of each ion is only due to the electrochemical potential gradient of the same ion.^4^ This assumes that off-diagonal elements in the Onsager coefficient matrix that account for coupling between fluxes are negligible.^80^ Likewise, Hodking and Huxley used the “independence principle” to explain how individual ions cross the membrane independently of the other ionic species present.^81^ In fact, several assumptions about independence at different levels (flux equations, diffusion coefficients, and constant field) are necessary to obtain either the permeability ratio of ion channels in the Goldman–Hodking–Katz (GHK) formalism or the transport number in ion-exchange membranes *via* the Teorell–Meyer–Sievers (TMS) model.^6^ Treatments based on ion independence consider the solution ideal, which simplifies their thermodynamic description, providing reasonable results in solutions with low ion density^28,82,83^ such as the range in which we observe non-linear scaling behavior here. However, moderately or highly concentrated solutions (as it could be the case in the proximity of charged membranes, or in certain locations inside nanoconfined channels^13^) must include effects such as long-range ion–ion correlations, solvent-excluded volume, dielectric friction during the movement of ions, ion advection by the fluid, or hard-core repulsion between ions.^83–87^ Here, these effects will not be considered for the sake of simplicity given that scaling behavior in concentrated solutions is linear and well described by available models.

Within the independence assumption, the equivalent circuit for ionic conduction must contain separate current branches for positive and negative ions arranged in parallel, as shown in the inset of Fig. 3B. Thus, the total conductance is given by:

7 *G* = *G*_+_ + *G*_−_

where8
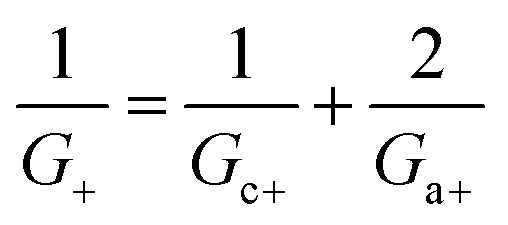
9
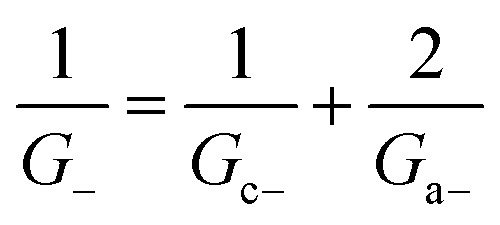


Charge effects due to pore charges *X*_c_ and membrane charges *X*_a_ can be introduced similarly to eqn (5) and (6), as follows:10
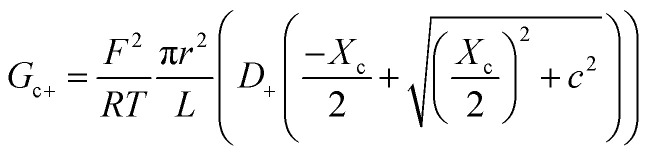
11
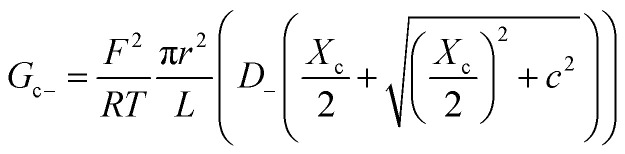
12
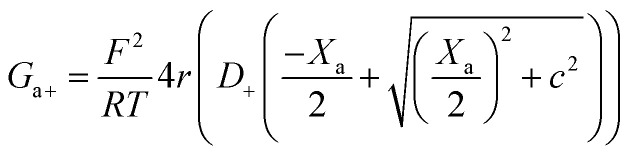
13
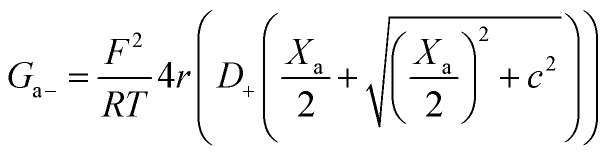


Being in parallel, the two branches share the total potential drop, but the current in each branch is determined by its total conductance (resistance). Accordingly, cations and anions carry different ionic currents when *X*_c_ ≠ 0 as expected from an ion selective channel^4^ or a permselective ion-exchange membrane.^6^

Predictions of this 2-branch (2-B) model are shown in Fig. 3B using the same parameters as for the 1-branch (1-B) model (Fig. 3A). The 2-B model gives identical results to the 1-B model in concentrated solutions (*G* scales linearly with *c*). Nonetheless, noticeable differences appear in dilute solutions. In neutral membranes, the 2-B model scales as *G* ∼ *c*^1^ just as the 1-B one, but yields smaller values of *G* by a factor that approaches 2 (see the ratio between models in Fig. S1†). For charged membranes, the 2-B model predicts contrasting outcomes depending on the sign of membrane charges. When *X*_a_ and *X*_c_ have the same sign, a current saturation *G* ∼ *c*^0^ is observed in the low concentration limit, in agreement with the 1-B model. However, when *X*_a_ and *X*_c_ have opposite signs, the conductance is largely inhibited in diluted solutions, being several orders of magnitude lower than the *G* predicted for neutral membranes. This suggests that lipid charges and protein charges operate separately and the interpretation of membrane permeability in terms of the net charge of the global system (lipids + protein) may overlook essential local effects.^38,88^ Indeed, electrostatic interactions between localized negatively and positively charged clusters are responsible for many observed phenomena in amphoteric surfaces, polyampholyte systems like gels, membranes and conducting polymers, polyelectrolyte multilayers^89^ and protein molecules in solution.^88^

Amazingly, for the case in which *X*_a_ > 0 (recall that we have considered *X*_c_ < 0) the 2-B model predicts an unprecedented scaling behavior *G* ∼ *c*^2^ in the low concentration limit. As far as we know, values *α* > 1 have never been reported theoretically (values are 0 ≤ *α* ≤ 1). The origin of the quadratic scaling *G* ∼ *c*^2^ lies in the fact that cations, which are counterions when *X*_c_ < 0, are also coions when *X*_a_ > 0. Hence, in the limit of low *c* of eqn (12) we find:14
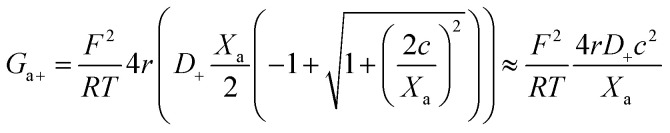
where we have used Taylor's expansion 
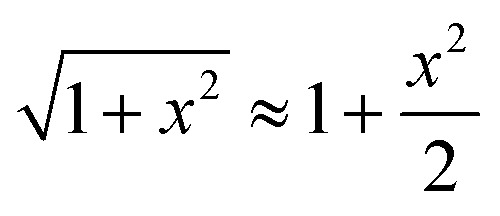
. Note that this scaling is impossible to achieve in the 1-branch model, which predicts the same behavior (*G* ∼ *c*^0^) regardless of the charge polarity when *X*_a_ ≠ 0.

Importantly, the scaling exponents *G* ∼ *c*^0^ and *G* ∼ *c*^2^ reported in Fig. 3B correspond to a cylindrical geometry and relatively high charge densities *X*_a_ in absolute values (100 mM). Considering a different channel shape (hourglass, fusiform, *etc.*) and/or smaller values for charge densities would imply that the limits *G* ∼ *c*^0^ and *G* ∼ *c*^2^ are attained in ultralow solution concentrations (micromolar or less) corresponding to the values of *G* below the experimental resolution. In practice, this means that, under the conditions that are experimentally accessible (millimolar range at the least), exponents show intermediate scaling in the low concentration regime with 0 ≤ *α* ≤ 1 when *X*_a_ and *X*_c_ have the same sign and 1 ≤ *α* ≤ 2 when the sign is opposite. Still, the 2-branch model can reproduce the experimental data reported here. Indeed, Fig. 3C and D correspond to the comparison of the 2-B model with the experimental points of OmpF and VDAC, respectively. The characteristic hour-glass shape of OmpF porin has been incorporated into the model by using an aperture of *r*_a_ ∼ 0.5 nm for the pore mouth and a smaller radius *r*_c_ ∼ 0.4 nm for the central constriction. In the case of VDAC, a cylinder of *r*_a_ = *r*_c_ ∼ 0.7 nm is used. The fitting parameters for OmpF (*X*_c_ = −250 mM, *X*_a_ = −50 mM PS, *X*_a_ = −5 mM PC, and *X*_a_ = 30 mM TAP) and VDAC (*X*_c_ = 200 mM, *X*_a_ = −100 mM PS, *X*_a_ = 0 PC, and *X*_a_ = 50 mM TAP) are in agreement with the existing literature.^47,48,56^

The channel effective charge can also modulate the scaling exponent, with a higher exponent for higher absolute *X*_c_ values. This is indeed what is observed experimentally (Fig. S2†) when OmpF channel negative effective charge is increased by increasing the solution pH.^47^ In positively charged TAP membranes, a supralinear scaling exponent *α* ∼ 1.3 is readily observed at higher concentrations (10–100 mM) compared to neutral pH, in which the scaling with *α* ∼ 1.4 is measured only for a very low salt concentration range (3–10 mM, inset of Fig. 2A).

### 3.3. Assessment of equivalent circuits with structure-based Poisson–Nernst–Planck calculations

We have demonstrated that the simplest approximation of an equivalent circuit is able to reproduce the experimental data, as long as it complies with the ion independence assumption. Still, it harbors many approximations that are worth analyzing by using more elaborated theoretical approaches. To begin with, equivalent circuits such as those in Fig. 3A and B are one-dimensional structure-less representations of a three-dimensional system that, in the case of complex objects like protein channels, represent a drastic simplification. Also, it is questionable how charge effects are introduced into an equivalent circuit *via* equilibrium concepts such as Donnan formalism together with the electroneutrality condition^21,90,91^ (note that Donnan equilibrium is obtained by imposing no ionic flux through the system, and its application here assumes that actual fluxes are small enough to justify a quasi-equilibrium situation).

To confront the equivalent circuit with a higher resolution approach, we use the mean-field equations that describe ion transport through membrane systems,^4–6^ known as the Poisson–Nernst–Planck (PNP-3D) model^92^ and implemented as described in detail elsewhere.^13,53^ We use the 3D atomic structures of OmpF and VDAC available at the Protein Data Bank (code 2OMF for OmpF and 3EMN for VDAC). Given each protein structure, the atomic charge was assigned to each atom according to the charmm36 force field and used as input for the PNP Python code based on the FiPy PDE solver.^93^ Ion fluxes and concentrations along the pore were calculated using bulk pH, salt concentrations, and electric potential at the channel entrances as boundary conditions.^53,54,68,94^ The existence of a charged membrane was simulated by adding a small charged region next to the ion-inaccessible membrane region.^13^ Ion diffusion coefficients were introduced as fitting parameters.

**Fig. 4:**
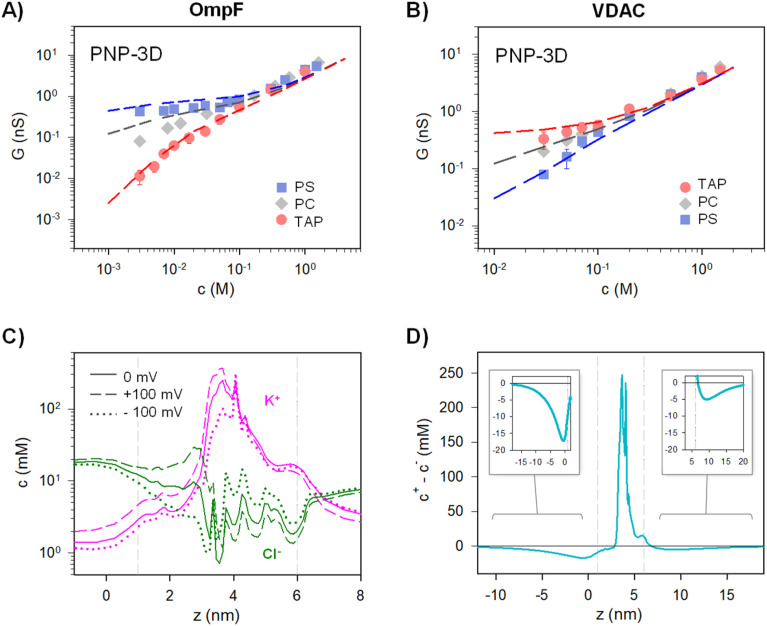
The PNP-3D model qualitatively reproduces the experimental conductance scaling of OmpF and VDAC. Conductance *vs.* concentration curves recorded with OmpF (A) and VDAC (B) (points) inserted in negatively (PS), neutral (PC) and positively (TAP) charged lipid membranes. Dashed lines correspond to the predictions obtained from the PNP-3D model. (C) Calculated concentration profile of cations (pink) and anions (green) across the OmpF longitudinal channel axis for equilibrium (*V* = 0 mV, solid lines) or under *V* = 100 mV (dashed lines) and *V* = −100 mV (dot lines). (D) Net concentration difference between cations and anions (*c*_+_ − *c*_−_) across the OmpF longitudinal access for the equilibrium condition (*V* = 0). The insets show an amplification of the indicated area. Graph titles are the same as in the original panel. In (C) and (D), calculations were performed using a positively charged membrane at 5 mM KCl. Vertical dashed lines indicate the limits of the membrane containing the protein.

*G versus c* curves obtained using PNP-3D calculations are shown in Fig. 4A and B and reproduce quite satisfactorily the experiments both in OmpF and VDAC for neutral (PC) and charged (negative (PS) and positive (TAP)) membranes. Interestingly, scaling behaviors obtained by PNP are very similar to those predicted by the 2-B model, particularly noteworthy is that *α* ∼ 1.4 in the low concentration limit of both OmpF in TAP and VDAC in PS. Such agreement between the 2-B circuit and the continuum models could be expected because the PNP equation system contains separate flux equations for cations and anions, so it intrinsically represents the same physical picture (ion independence) of the 2-B model in Fig. 3B.

PNP-3D calculations allow probing another assumption of the equivalent circuits, namely the fact that the equilibrium ionic concentrations could approximately account for non-equilibrium currents. Starting from the 3D data, we averaged the concentration of ions across the cross-sectional accessible area along the pore axis (see ref. 53 for details) to obtain a concentration profile under different conditions (with and without applied voltage). Fig. 4C shows the calculated concentration profile of cations (pink) and anions (green) along OmpF pores for equilibrium (solid lines) or under *V* = 100 mV (dashed lines) and *V* = −100 mV (dot lines) for a positively charged membrane at 5 mM KCl. Although small differences are evident when voltage is applied, no dramatic changes are observed between equilibrium and non-equilibrium average concentrations. For instance, the average concentration of cations inside the channel region (vertical dashed lines indicate the limits of the membrane containing the protein) changes from ∼48 mM in equilibrium to ∼62 mM for *V* = 100 mV and to ∼38 mM for *V* = −100 mV. For anions, the change is less evident (4 mM < *c* < 8 mM). For calculations performed at 100 mM (Fig. S3†), the effect of applied voltage is even less important, and average concentrations under applied voltages differ from equilibrium values by less than 10%. Note also that the assumption of quasi-ohmic behavior for the studied channels is supported by experiments as *I*–*V* curves for both OmpF and VDAC are approximately linear at all concentrations studied for a wide range of applied voltages (Fig. S4†). Therefore, we can conclude that the equilibrium approximation used in the equivalent circuit model is reasonable.

The use of a logarithmic scale in Fig. 4C facilitates the simultaneous observation of cations and anions but exaggerates the contribution of lipid charges in the channel mouths. For this reason, in Fig. 4D we display the net difference between cations and anions (*c*_+_ – *c*_−_) for the equilibrium condition (*V* = 0). Interestingly, Fig. 4D suggests that the charge imbalance is not restricted to the region where membrane and channel charges are located (region between grey vertical dashed lines), but a small imbalance is extended considerably far away in the solution (∼10 nm). This means that the step-like potential and concentration profiles characteristic of Donnan/electroneutrality assumptions are crude oversimplifications of the actual ones. Still, the approximation is acceptable here, given that the charge imbalance extending out of the channel/membrane in Fig. 4D is small. In line with our results, previous studies concluded that Donnan potentials derived without any assumptions agree with the simplified treatment (zero ionic flux and local electroneutrality) for typical protein channels embedded in biological membranes (∼5 nm) except for ultrashort pores (<2 nm).^91^ For these short pores, the discrepancies arise precisely from the fact that some parts of these channels are never electroneutral^91^ just as shown by a series of more recent studies.^10,21,95^ Of note, MD simulations in ion-exchange systems show that, despite the lack of atomic detail, the Donnan formalism is accurate enough to justify its application to systems with nanometer-sized pores.^96^

**Fig. 5:**
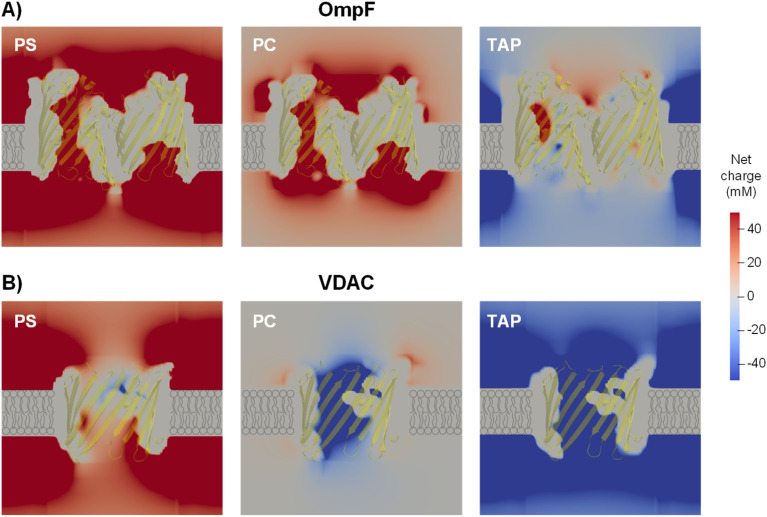
An excess of net mobile charges extends into the solution surrounding the channel. PNP-calculated 3D maps of the OmpF (A) and VDAC (B) channels embedded in a negative (PS), neutral (PC) or positive (TAP) membrane at 5 mM KCl and *V* = 0 showing the system net charge. The overall system (protein + membrane + solution) is electroneutral. Cartoon-style membrane lipids are drawn to indicate the location of the lipid bilayer. The proteins are shown in light yellow. For OmpF, one of the monomers is not shown for clarity.

To address more accurately the issue of charge neutrality, one may use the three-dimensional information provided by the 3D PNP. Fig. 5 shows the 3D maps of the OmpF (A) and VDAC (B) channels embedded in neutral (PC), negatively charged (PS) and positively charged (TAP) membranes at 5 mM KCl and no applied voltage. The overall system (protein + membrane + solution) is electroneutral. For OmpF in PC, negatively charged residues outnumber the positive ones so that the overall protein is selective to cations. Accordingly, there is an excess of net positive mobile charges (light red) that extends into the solution. As could be expected from a 3D object, the screening of protein charges exerted by mobile ions occurs in all spatial directions, including the solution inside the pore and outside the protein/lipid physical boundaries. When the neutral membrane is replaced by a negatively charged (PS) one, the excess of net positive mobile charges increases and extends further into the solution. However, when the membrane is positive (TAP), the net charge of mobile ions becomes mostly negative (light blue) except in small spots in red, revealing the existence of separate charge clusters as those hypothesized in the equivalent circuits. Also, the total charge concentration inside and around the channel is clearly reduced (leading to a reduced conductance and shown in Fig. 4A). For the case of VDAC (Fig. 5B) in PC, the image is similar to that of OmpF in the same membrane, but with an excess of net negative charge slightly extending into the solution and making the overall protein selective to anions. When the membrane is charged, the effect is similar to that of OmpF in absolute values, but with opposite polarity: the excess of negative net charges increases and extends further into the solution for TAP while total charge concentration in and around the channel is reduced in PS. Altogether, our results show that membrane lipids and protein residues could act as separate interacting clusters of charge, yielding a remarkable decrease in channel conductance not anticipated in classical models in which only the overall net charge is considered. Also, the existence of diverse regions in which there is an excess of charged mobile ions evidences that local electroneutrality could be a reasonable assumption for the description of transport processes that occur in regions considerably larger than the corresponding Debye length,^75,97^ but this assumption fails in smaller regions such as at the membrane–solution interface or the interior of an electrical double layer, as shown in Fig. 5. A more in-depth analysis considers the three length scales that govern the charge regulation in nanopores: the Debye length (ion–ion interactions), the Gouy–Chapman length (ion–wall interactions) and the pore diameter.^10^ Interestingly, this study predicts a regime of low surface charge and small salt concentration where electroneutrality is likely to be broken depending on the pore size.

## 4. Conclusions

By measuring *G*–*c* relationships in two biological ion channels reconstituted in lipid bilayers of varying charge, we obtain a variety of scaling behaviors *G* ∼ *c*^*α*^, including never reported before scaling exponents *α* > 1. On the basis of these findings, we demonstrate that equivalent circuits should include separate contributions for each ionic current to account for experiments. This implies that the overall description should include both “access/channel” and “cation/anion” paradigms to explain the interplay between inner and outer surface charges. Indeed, unprecedented scaling exponents *α* > 1 can only be explained when counterions within the pore are also coions for the charged membrane in the pore mouth.

Besides, we show that simplified continuum models, which consider electroneutrality and Donnan equilibrium, yield extremely similar scaling behavior to the structure-based Poisson–Nernst–Planck equations computed without those simplifying assumptions. Therefore, intermediate scaling exponents 0 ≤ *α* ≤ 1 and/or 1 ≤ *α* ≤ 2 do not correspond to deviations from classical Donnan/electroneutrality assumptions, but rather to the structural features of the pore such as non-cylindrical shape, inhomogeneous charge distribution or entrance effects.

In contrast to the predictions of widely accepted models, we show that charged membranes can significantly reduce the conduction of ion-selective channels in dilute solutions when lipid charges are of the same sign as the channel intrinsic selectivity (VDAC selective to anions/negative DOPS and OmpF selective to cations/positive DOTAP). These cases correspond to situations in which membrane charges and channel net charges act as separate clusters of opposite charge, creating a system that cannot be understood in terms of the overall net charge.^88^ Thus, negatively charged membranes could substantially decrease the conduction of anion selective channels in diluted solutions, a realistic situation in the intracellular space of negatively polarized cells in which mobile anions are excluded and their concentration is extremely low (∼5–10 mM)^98^ in comparison with the extracellular fluid where concentration is much higher (∼150 mM). Hence, estimations of the number of open channel cells made from permeability measurements^99^ could have diverse interpretations if channel conductance *G* is extrapolated from concentrated solutions to dilute ones assuming a wrong scaling behavior.

Because positively charged membranes do not exist in a biological environment,^100^ we cannot expect this supralinear conductive mechanism to be effective for actual cation-selective biochannels such as OmpF or many others. However, the importance of our results goes beyond the academic rationalization of the pore conductance paving the way for nanofluidic devices based on the independent functionalization of inner and outer channel surfaces.^40^ Within this methodology, not only charge modification could be managed, but also surface wettability could be adjusted *via* hydrophobic interactions and detection performance could be enhanced by modifying probes.^39^ We note that many of these features could be attained, at least for exploratory purposes, in biological ion channels reconstituted in membrane systems (planar bilayers, liposomes, *etc.*) whose composition could be fine-tuned at will.

Finally, we would like to point out that our results provide a unified framework to study ion transport in confined geometries, while highlighting the fact that scaling arguments are powerful yet simple tools to provide a comprehensive perspective on various pore-forming systems. This is particularly relevant for two contrasting types of objects: on the one hand, channels with highly complex structures that require significant computational resources to perform atomistic simulations or even continuum theories taking all effects into account and on the other hand, systems whose actual structure is unknown such as proteolipidic channels (*i.e.* peptides, toxins, and viroporins) or abiotic nanopores with inhomogeneous geometry and/or charge distribution.

## Conflicts of interest

There are no conflicts to declare.

## Supplementary Material

NA-006-D4NA00540F-s001

## Data Availability

All data that support the experimental findings are included within the article. The data that support the theoretical findings are available from the corresponding author upon reasonable request.
