# Supplementary material for: Supralinear scaling behavior of ionic transport in membrane nanochannels regulated by outer-surface charges
Source: Nanoscale Adv. 2024 Oct 14;6(24):6344–57. doi: 10.1039/d4na00540f (PMC11515935; doi:10.1039/d4na00540f)
Supplement: NA-006-D4NA00540F-s001 [file NA-006-D4NA00540F-s001.pdf]

Supplementary Information (SI) for Nanoscale Advances.
This journal is © The Royal Society of Chemistry 2024

Supporting information for manuscript

Supralinear scaling behavior of ionic transport in membrane nanochannels regulated by outer-surface charges

Laidy M. Alvero-Gonzalez[1], Marcel Aguilella-Arzo[1], D. Aurora Perini[1,2], Lucie A. Bergdoll[3], María Queralt-Martín[1]*, Antonio Alcaraz[1]*

[1]Laboratory of Molecular Biophysics. Department of Physics. University Jaume I, 12071 Castellón, Spain

[2]Instituto de Ciencia Molecular, Universidad de Valencia, Catedrático José Beltrán-2, 46980 Paterna, Spain

[3]Laboratoire d'Ingénierie des Systèmes Macromoléculaires, CNRS - Aix Marseille Université, 31 Chemin Joseph Aiguier, Marseille France

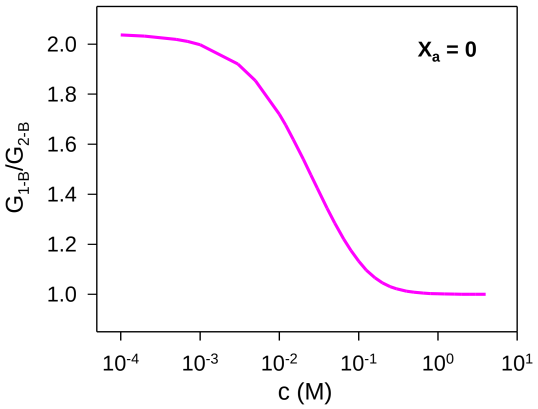

Figure S1. Ratio between conductances obtained with the 1-branch (1-B) and the 2-branch (2-B) model for a neutral membrane. Parameters used are as in Figure 3A and 3B.

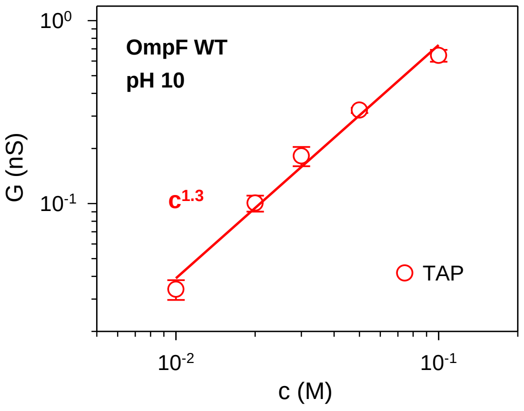

Figure S2. Conductance vs. concentration curve (open circles) measured with OmpF WT inserted in a positively (TAP) charged lipid membrane at pH 10 in the KCl concentration range of 10-100 mM. The solid line is a fit of G ~ c^{α} yielding supralinear scaling (α ~ 1.3).

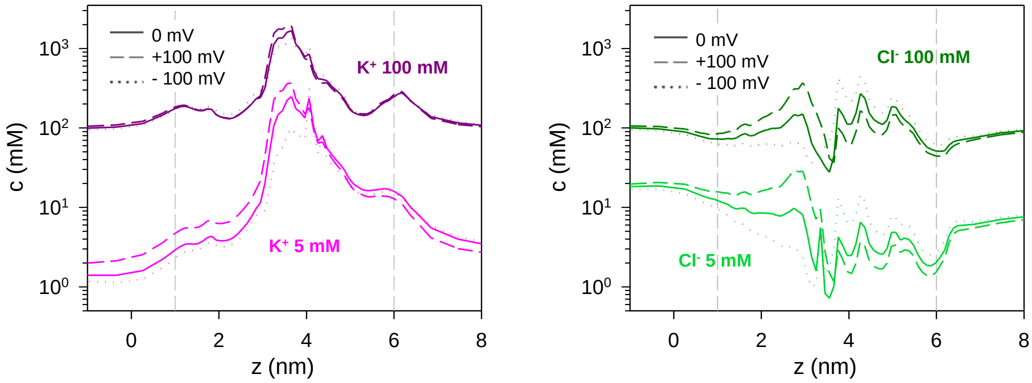

Figure S3. Calculated concentration profile of cations (pink, left panel) and anions (green, right panel) across the OmpF longitudinal channel axis for equilibrium (V = 0 mV, solid lines) or under V = 100 mV (dashed lines) and V = -100 mV (dot lines). Light-colored and dark-colored lines correspond to c = 5 mM and c = 100 mM KCl, respectively.

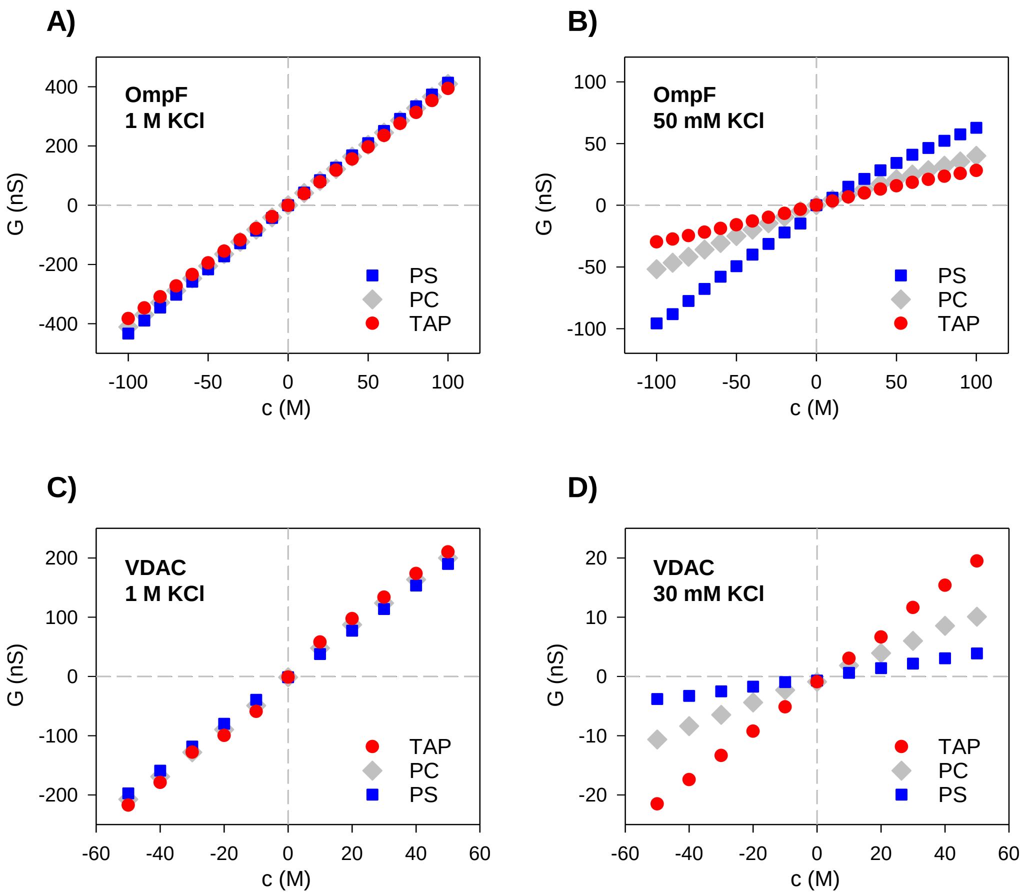

Figure S4. Example I-V curves measured for OmpF and VDAC at high and low KCl concentrations inserted in neutral (PC, grey diamonds), positively charged (TAP, red circles) and negatively charged (PS, blue squares) lipid bilayers: **(A)** OmpF in 1 M KCl; **(B)** OmpF in 50 mM KCl; **(C)** VDAC in 1 M KCl; **(D)** VDAC in 30 mM KCl; I-V curves are approximately linear in all cases. The voltages shown are those applied to measure channel conductance during the voltage-clamp experiments in all salt concentrations explored: from 0 to ±60 mV for VDAC and from 0 to ±100 mV for OmpF. Higher voltages were only applied to confirm single-channel insertion and check channel closure (see Materials and Methods for more information).
